# Unexplored Mechanisms of Photoprotection: Synergistic Light Absorption and Antioxidant Activity of Melanin

**DOI:** 10.3390/antiox14040376

**Published:** 2025-03-21

**Authors:** Arianna Menichetti, Dario Mordini, Silvia Vicenzi, Agata Pane, Marco Montalti

**Affiliations:** 1 Department of Chemistry “Giacomo Ciamician”, via Francesco Selmi 2, 40126 Bologna, Italy; arianna.menichetti2@unibo.it (A.M.); dario.mordini2@unibo.it (D.M.); sivia.vicenzi4@unibo.it (S.V.); agata.pane2@unibo.it (A.P.); 2 Tecnopolo di Rimini, via Dario Campana 71, 47922 Rimini, Italy

**Keywords:** polydopamine, ROS, nanoparticles, ultrafast transient absorption, radical scavenging, light scattering, singlet oxygen, cosmetics, solar light, UV light

## Abstract

Light exposure has relevant effects both on living organisms and artificial materials. In particular, ultraviolet radiation is known to kill living cells and damage human skin but also degrade important artificial materials like plastics. In nature, the main pigment responsible for photoprotection is melanin, which is able both to prevent penetration of light by absorption and scattering and to block the action of light-generated radicals thanks to its antioxidant properties. The combination of light extinction with antioxidant action is still the most diffused and effective approach to photoprotection. Nevertheless, up to now, these two mechanisms, light extinction and antioxidant activity, have been considered independent. Recent studies showed that exposing melanin to light leads to an increase in its radical content and possibly in its antioxidant activity. Do light extinction and antioxidant activity work in synergy for photoprotection in nature? In this paper, we discuss the steps still needed to answer this intriguing question.

## 1. Introduction

Exposure to light, and in particular to ultraviolet (UV) radiation, can kill living cells [1]. The effect of solar light on human skin is indeed multifaced, and it involves a complicated combination of processes [2]. Nevertheless, it is accepted worldwide that excessive exposure to solar light can be dangerous both in the short (months to few years) and long (decades) timescale [3]. For this reason, several approaches have been developed to protect human skin from the negative effects of solar light. A very relevant one is the incorporation, in cosmetic products, of chemical agents able to protect human skin [4,5,6,7].

**Figure 1:**
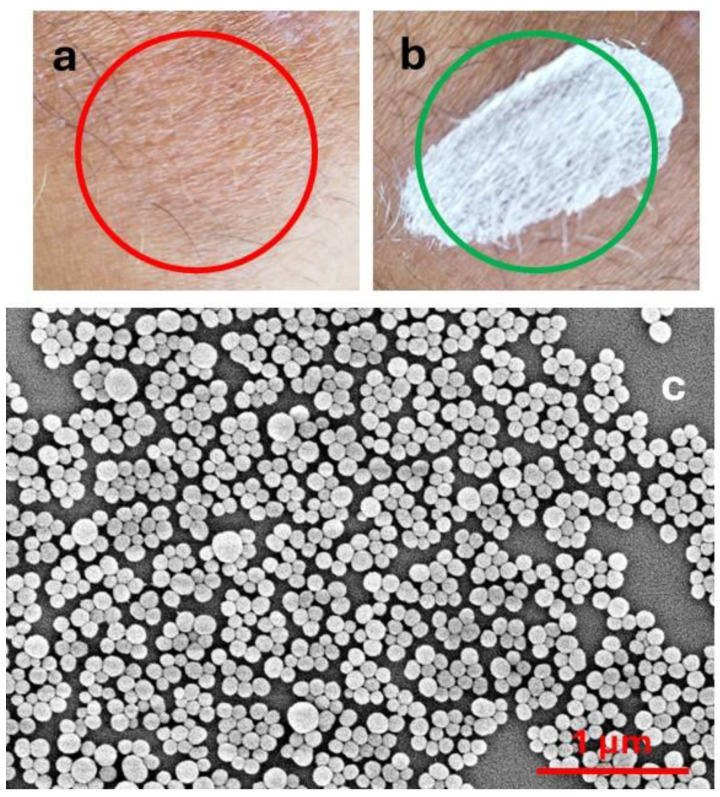
(**a**) Sunscreen containing TiO_2_ nanoparticles; (**b**) sunscreen containing ZnO microparticles. (**c**) SEM image of melanin NP from cuttlefish ink.

The action of these products, which are typically applied to the skin in the form of creams, sprays, sticks, etc., exploits the combination of two effects: (a) light extinction [8] and (b) antioxidant activity [9,10,11]. Light extinction shields the skin from light. This can be achieved either by light absorption or by light scattering. The actual result of the two processes is very different. In particular, light absorption produces excited states and hence potential photo-reactivity or, in the case of complete non-radiative deactivation, heat photogeneration, which can damage skin cells. Light scattering is efficient only for particulate filters, and it does not produce excited states. On the other hand, light scattering is strongly size-dependent and is wavelength selective only for very small particles (Rayleigh scattering). As a consequence, in order to have selective scattering in the UV, and hence products transparent in the visible range (Figure 1a), as typically requested by the market, particles with diameters of tens of nanometres (nanoparticles, NPs) are necessary [12,13,14]. Products based on microparticles, on the contrary, are typically whitish even after drying (Figure 1b). It is very interesting to consider that the natural material responsible for photoprotection, melanin, is able to absorb and scatter light, being present in the form of NPs in nature (Figure 1c) [15,16].

**Figure 2:**
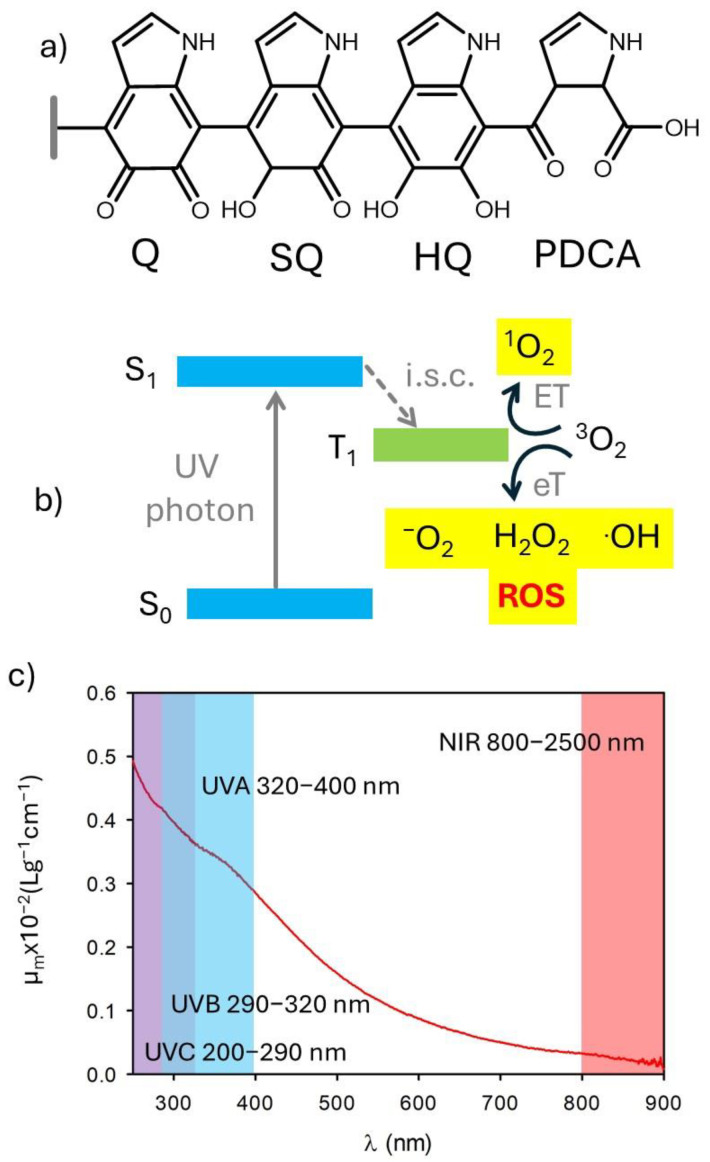
(**a**) Scheme of the generation of reactive oxygen species (ROS) upon UV irradiation either of photoprotective molecules or intrinsic chromophores of the skin. (**b**) Possible chemical structure of melanin showing the presence of di-hydroxyquinones (HQ), semiquinones (SQ), and quinones (Q). Oxidation of Q leads to the formation of pyrrole 2,3 dicarboxylic acid (PDCA). (**c**) Extinction spectrum of polydopamine nanoparticles. Adapted with permission from ref. [27].

Several kinds of NPs and organic molecules are known to generate reactive oxygen species upon light irradiation [17,18,19,20,21]. This also occurs in the case of species used for photoprotection and chromophores present in the skin [22]. The use of antioxidants in products for photoprotection is mostly the result of two needs [23]: (i) preventing the formation of potentially dangerous reactive species, including reactive oxygen species (ROS), upon excitation by photo-protecting filters (the process of ROS generation is schematized in Figure 2a), and (ii) quenching the reactive species formed due to the absorption of visible and NIR light by the skin chromophores (Figure 2a). In this context, it is interesting to note that melanin shows important antioxidant activity [24,25,26,27]. This antioxidant activity has been attributed to the presence of exposed orthoquinone units (Figure 2b) [28].

In this paper, we want to stress that studies on both natural and biomimetic melanin showed not only that melanin is able to absorb light but that this process increases the radical content of melanin itself [29]. Hence, irradiated melanin is expected to be a better antioxidant than melanin in the dark. This hypothesis, which still requires definitive confirmation, suggests that light extinction and antioxidant activity may work in nature in a synergistic way, suggesting new, intriguing mechanisms for photoprotection and, more generally, for the design of photoactive nanomaterials.

In this review paper, we focus on recent and relevant papers treating light extinction and antioxidant properties of biomimetic and natural melanin, whose main chemical properties are discussed in Section 2. Optical and antioxidant properties of melanin are typically analysed independently in the scientific literature. For this reason, we will discuss them into two different sections (Section 3 and Section 4, respectively). As far as optical properties are concerned, a detailed analysis of the photophysical processes occurring in melanin upon excitation was allowed by recent studies based on ultra-fast transient absorption (UFTA) spectroscopy. Regarding the antioxidant activity, the results strongly depend on the technique used for its determination. In general, it is recognized that melanin exhibits antioxidant and radical scavenging activity. Nevertheless, quantification of this activity is not univocal and is strongly affected by the method applied. Even more debated is the effect of irradiation on these antioxidant properties. In fact, while it is recognized that light exposure produces an increase in the radical character of melanin, it is not completely clear if this change leads to an improvement or to a worsening of the antioxidant activity. This relevant point is discussed in Section 5. Given the relevance of experimental methods in determining the antioxidant activity of melanin, the different possibilities are summarized in Section 6, where the most critical issues related to the application of the most common methods under light irradiation are considered, and suggestions are offered for future developments.

## 2. Natural and Biomimetic Melanin

**Figure 3:**
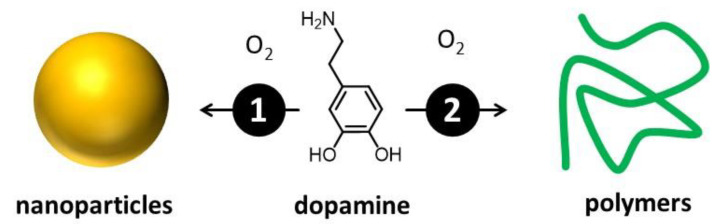
Oxidative polymerization of a molecular precursor—in this case, dopamine—leads to the formation of products with different levels of cross-linking. As a consequence, both small, water-soluble, poorly cross-linked polymers and nanoparticles are formed. The two species present not only different morphology but also different optical and chemical properties.

The actual composition and structure of melanin are still not completely known [16,30,31]. This is mainly due to its complexity, which arises from the simultaneous presence of different molecular units that can be difficult to isolate and identify [32]. Nevertheless, melanin-like materials have found applications in fields of high social and economic impact, including medicine [33], energy conversion and storage [34], catalysis [35], and materials science [33]. What is widely accepted is that melanin results from the oxidation/polymerization of one or a few molecular precursors via an enzymatic process [34]. For this reason, different kinds of melanin have been mostly classified on the basis of the molecular precursor [36]. Messerschmidt and Lee demonstrated that a biomimetic analog of melanin, polydopamine (PDA) [37], can be used to coat almost any kind of surface, allowing easy functionalization [38,39]. PDA is extremely biocompatible, and the Gianneschi group demonstrated that PDA NPs are taken up by human keratinocytes just like melanosomes, and they perform analogous photoprotective functions [1,40,41]. Thus, biomimetic melanin has been demonstrated to closely resemble its natural counterpart and serves as a representative natural model. A key advantage is that biomimetic melanin can be easily prepared and purified in the laboratory in relevant amounts through environmentally friendly and extremely simple processes [27]. On the contrary, extraction and purification of natural melanin can be difficult, time-consuming, and expensive, and it yields low amounts [42]. From the technological point of view, even more importantly, biomimetic melanin can be easily modified during the synthesis, offering a unique versatility [43,44]. We would like to stress that melanin can exist either in the form of nanoparticles or water-soluble small polymers. These materials, as schematized in Figure 3, can derive from the same precursor and the same chemical process, yet exhibit very different optical and chemical properties [27]. If this variability complicates the actual definition of the different kinds of melanin, which, as mentioned, is mostly based on the nature of the molecular precursor [36], it also demonstrates the actual versatility of these materials, which display easily adjustable optical, chemical, and morphological features [16].

## 3. Light Absorption by Melanin

Broadband absorption, ranging from UV to NIR, is one of the typical properties of both natural and biomimetic melanin (Figure 2) [1,27,41,45]. Environmental and dermatological photobiologists further divide UV into UVA (320–400 nm), UVB (290–320 nm), and UVC (200–290 nm), since the different wavelengths have very different effects on human skin and living organisms (Figure 2) [45]. Moreover, the different UV wavelengths are filtered with different efficiency by the atmosphere. A constant “sun” is termed to describe an average value of irradiance, outside the atmosphere, which is 1.37 kW m^−2^. Of this, about 9% is in the ultraviolet range (λ < 400 nm). On the other hand, the UVC component is completely extinguished by the atmosphere, and UV radiation on the earth’s surface is roughly composed of 6% UVB and 94% UVA. As shown in Figure 2, melanin absorbs efficiently both UVA and UVB.

**Figure 4:**
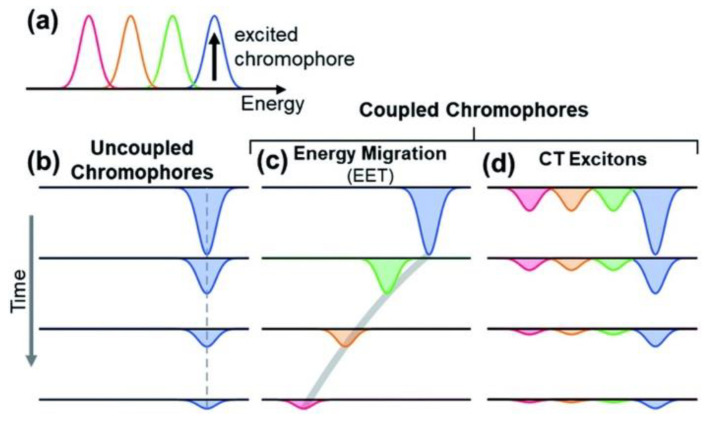
Scheme of the possible origins of the broadband spectrum of melanin. Multiple transitions are observed (**a**). These transitions may be localized on different chromophores, which may be non-interacting (**b**) or be involved in energy-transfer processes (**c**). As an alternative, some transitions may be delocalized, involving optical electron transfer between the chromophores (**d**). Different colors have been used to represent different transitions at different energies. Reproduced with permission from ref. [46].

**Figure 5:**
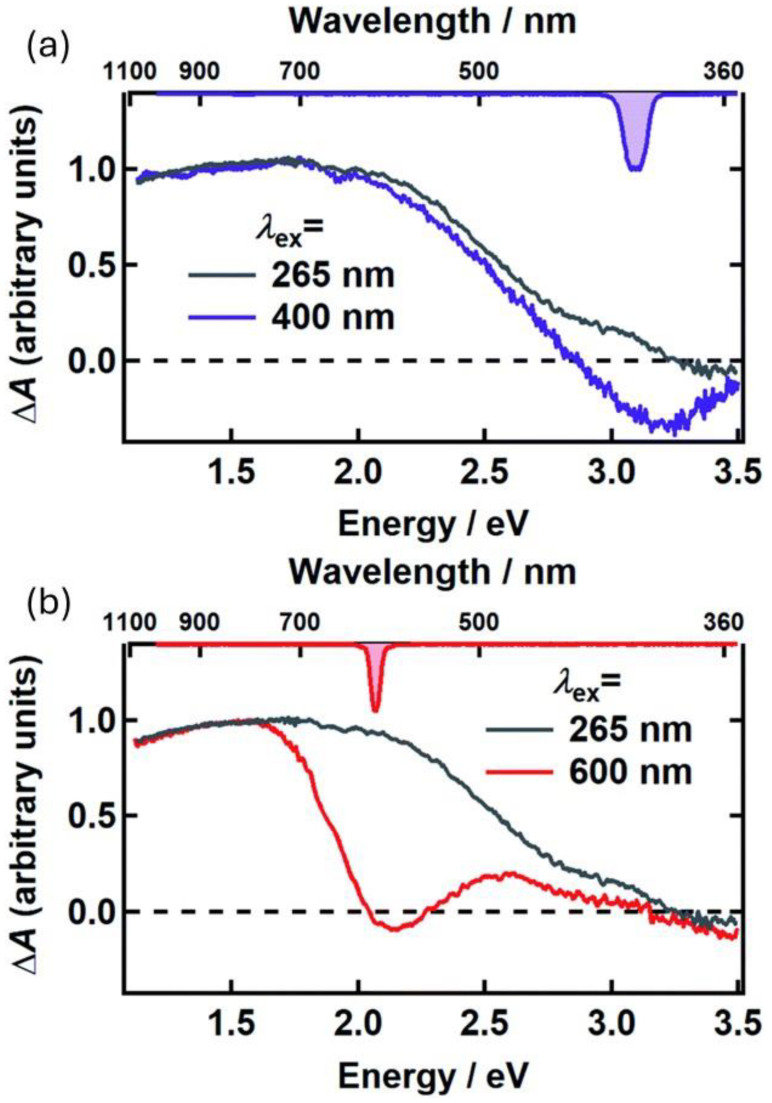
UFTA spectra observed 500 fs after excitation, for poly-L-DOPA upon excitation at 400 nm (**a**) and 600 nm (**b**). In both cases, these spectra were compared with the UFTA spectrum upon excitation at 265 nm. Spectra reveal hole-burning at the excitation wavelength, as well as a broad signal clearly detectable in the red-NIR region (600–900 nm). Adapted with permission from ref. [46].

**Figure 6:**
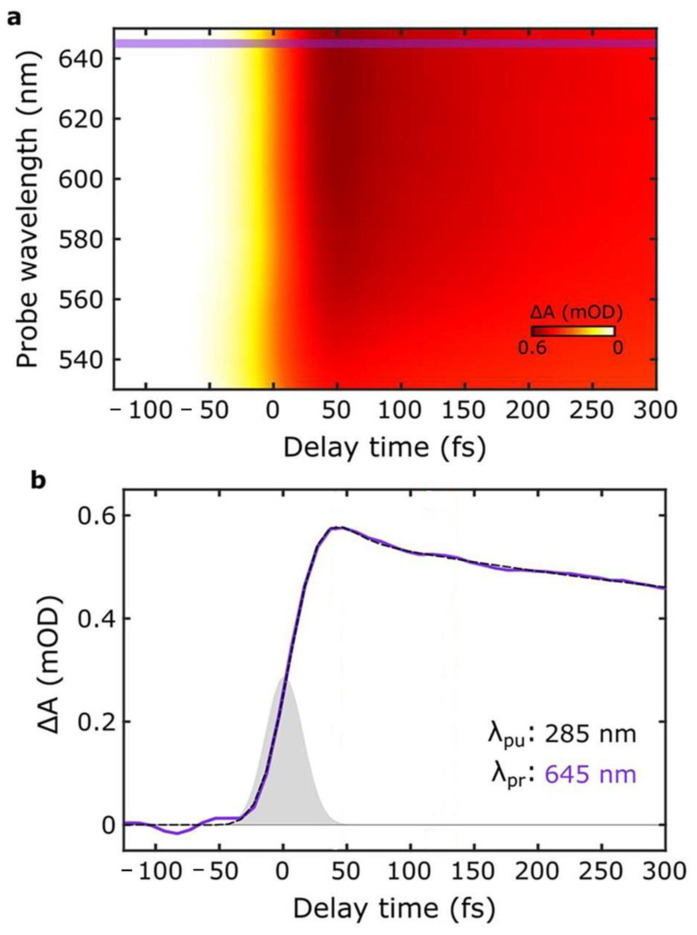
(**a**) UFTA spectrum observed for PDA NPs upon excitation at 285 nm. Violet lines identify 645 nm (**b**) Kinetics of the signal at 645 nm upon excitation at 285 nm (violet line, black dashed line is the fitting). This signal does not arise from hole-burning and has been identified as a charge-transfer transition. Adapted with permission from ref. [48].

As mentioned, the protection of living cells from the effects of light requires not only that light is prevented from reaching the cells themselves but also that non-dangerous species are formed upon light absorption. For this reason, the excited-state deactivation dynamic of melanin and related materials has been investigated by different groups via ultra-fast transient-absorption (UFTA) spectroscopy [46,47,48]. These studies focused on the kinetics of deactivation of photoexcited melanin and on understanding the origin of the broad absorption band of melanin and related materials. Two main models have been proposed to explain the nature of the transitions in melanin-like materials: (i) According to the first model (chemical complexity), optical transitions in melanin are due to the presence of several different chromophores, each of which absorbs in a specific region of the spectrum (Figure 4a–c). Hence, at different excitation wavelengths, different chromophores would be excited. (ii) The alternative model considers the presence of electron-donor and electron-acceptor chromophores that undergo optical charge-transfer transitions (Figure 4d), resulting in transitions that are delocalized across different chromophores. The former model was used by Kholer and colleagues to explain the photophysical behavior of poly-L-DOPA [46]. In more detail, these authors observed a phenomenon known as hole-burning, which is typical of a set of non-communicating chromophores (Figure 4b) that are excited individually and do not undergo energy-transfer processes (Figure 4d). We would like to stress that, beyond their focus on the hole-burning effect, the authors also observed broad transient absorption spanning from the UV to the NIR upon localized excitation, as shown in Figure 5. Results reported by Montalti, Maiuri, and Cerullo confirm these observations: a combination of localized transitions, leading to hole-burning, and of charge-transfer non-localized transition were observed (Figure 6) [48]. The main difference between the two works by Kholer and Cerullo lies in the relative contribution of localized and charge-transfer transitions (which is dominant in the work by Cerullo). We believe this difference is due to a significant difference in the sample analyzed, obtained by polymerization of L-DOPA in the former case and by dopamine in the latter. The effect of aggregation and inter-chromophoric interactions in broadening the absorption spectrum of melanin was also demonstrated through UFTA measurements by Warren and colleagues [47].

We would also like to stress that the presence of charge-transfer interactions underlying the optical properties of melanin were recently exploited to control its spectral features and extend the absorption band into the NIR region [49]. In this framework, melanin can be considered as an organic semiconductor where transition involves electron transfer from electron-donor dihydroxiquinone units to electron-acceptor quinones, a mechanism that justifies the attribution of the broad absorption to charge-transfer delocalized transition.

Regarding the kinetics of the excited-state deactivation, all authors agree that it is quite fast (a few picoseconds’ lifetime) and almost completely nonradiative [46,47,48]. This observation is very important in consideration of the photoprotective action of melanin, since it demonstrates that absorbed light energy is dissipated fast without forming potentially dangerous reactive species or species that can react diffusely with oxygen. Some studies, on the other hand, seem to be in contrast with this lack of photoreactivity [50]. This apparent inconsistency is due to the fact that UFTA can only detect the dominant processes occurring on short timescales, making it difficult to identify processes with very low quantum yields (<1%). Indeed, the photoactivity of melanin is very modest; for example, the photo-consumption of oxygen by melanin has been reported to have a quantum yield of ~0.1% [51].

## 4. Antioxidant Activity of Melanin

**Figure 7:**
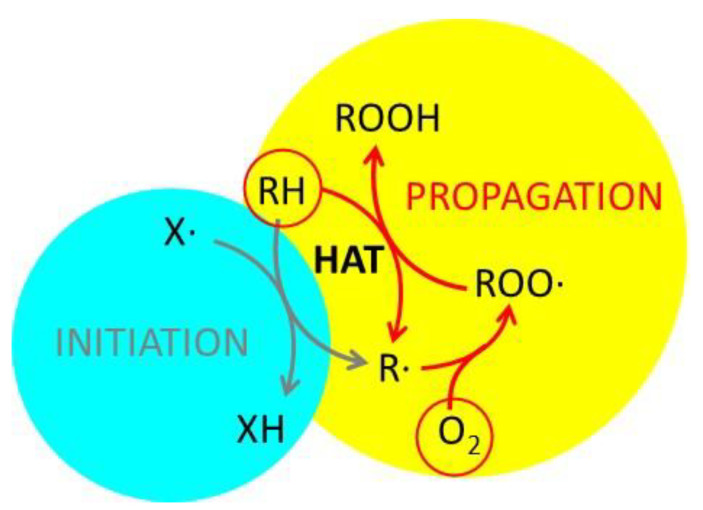
Schematic representation of an oxidative process. The organic precursor RH is oxidized by atmospheric oxygen to form ROOH. The propagation mechanism involves the formation of the radical ROO• and a step of hydrogen atom transfer (HAT). The overall process is initiated by the radical X•. Adapted from ref. [64].

Although radical scavenging and antioxidant activity are often confused, they are very different [52,53,54,55]. In simple terms, antioxidant activity is the ability of a substance to prevent or slow down the oxidation of an organic precursor by an oxidant, typically atmospheric oxygen [56,57,58]. Although various oxidative processes can be considered, some of them are of fundamental importance for life [59]; indeed, aging itself has been considered an oxidative process [3,60]. They also play a significant role in technology, as oxidation leads to food and materials degradation [61]. These oxidative processes are generally quite slow, but they can be greatly accelerated by small amounts of radicals (initiators), which simply act as catalysts triggering the radical propagation schematized in Figure 7. This scheme has been proposed by several research groups to describe the role of radicals in oxidation [62,63,64].

The ability to prevent oxidation can arise either from inhibiting the initiation or the propagation step. In both cases, the quenching of radicals is required; thus, the ability to quench radicals is expected to lead to a strong antioxidant activity, and radical quenching and antioxidant activity are sometimes confused. However, radicals involved in the initiation (X•) and in the propagation (ROO•) process are very different, and a chemical species able to quench X• may not be able to quench ROO•. As a consequence, radical scavenging activity should be referred to specific radicals and cannot be simply correlated with antioxidant activity [52].

Several studies have revealed the ability of different kinds of melanin to scavenge radicals and act as antioxidants [26,41,64,65,66,67]. A detailed discussion of the antioxidant properties of melanin is reported in a dedicated review paper [26]. Interesting results were reported by Gianneschi’s group, who investigated the radical scavenging ability of melanin obtained from dopamine and compared it with allomelanin [40]. These authors demonstrated that the effective radical-scavenging activity of polydopamine was overtaken by allomelanin. The mechanism involved in the antioxidant activity of allomelanin was also recently investigated [64]. In a recent work, it was also demonstrated that the radical-scavenging activity of polydopamine strongly depends on the size and morphology being much greater for small polymers than for large nanoparticles [27]. The mechanism responsible for the antioxidant activity of polydopamine has also been investigated [28]. Owing to these properties, melanin has been proposed as an anti-inflammatory agent in medicine, e.g., for wound healing [25] or in periodontal disease [68]. A major advantage in comparison with other antioxidants is that melanin has been reported to be active against all the known reactive oxygen and nitrogen species (RONS) formed in oxidative stress situations, including •O_2_^−^, H_2_O_2_, •OH, •NO, and ONOO^−^ [69].

The interaction of dopa, 5-S-cysteinyldopa-melanins, and a natural melanin with oxidizing (•OH, •N_3_) and reducing (e^−^_aq_, •CO_2_^−^) radicals, generated by pulsed radiolysis, was investigate in phosphate buffer (pH = 7.4) by Sarna et al. [70]. In particular, changes in the absorbance of melanin or of the radicals were detected over time. Interestingly, very different behaviors were observed in the absorbance of melanin when comparing the effects of oxidizing versus reducing radicals: an increase in the absorbance in the visible spectrum was observed in the former case, while a decrease was seen in the latter. This difference is partially in contrast with the observation that oxidation of melanin with strong oxidizers leads to melanin bleaching. Indeed, we believe this is consistent with the complexity of the multi-step chemistry of melanin. Formation of melanin, and the appearance of its typical broad, unstructured absorption band, is the result of oxidation of non-colored molecular precursors (e.g., dopamine, [71]). In this context, oxidation results in darkening and reduction in bleaching. On the other hand, excessive oxidation of melanin leads to the formation of small, non-colored molecules like pyrrole-2,3,4,5-tetracarboxylic acid [72]. According to Sarna et al., •OH shows the highest reactivity with melanins, confirming the efficient antioxidant activity of these species. The excess of reduced groups with respect to oxidized ones in melanin models was confirmed by Rozanowska et al., who also applied pulsed radiolysis [73]. Burke et al. showed that melanosomes in cells subjected to chemically induced oxidative stress provide cytoprotective function by acting as antioxidants [74]. Kakzara et al. demonstrated that, in contrast to what has been supposed, complexation of metals like iron by melanosomes is not primarily responsible for this antioxidant activity, which is quite independent on the actual amount of iron in the cells [75].

## 5. Light-Enhanced Radical Content in Melanin

Both natural and artificial melanin are known to contain some radical content, clearly detectable by EPR. According to Meredith and colleagues [29], in the case of eumelanin, two radical species indeed are simultaneously present: (i), the semiquinone radical (SQR), whose signal dominates the spectrum (g = 2.004) in suspension. This EPR signal is photoactive and is reversibly affected by pH, metal ion chelation, and temperature. (ii) The second is a carbon-centered radical (CCR), which dominates the solid-state EPR signal (peak feature at g = 2.0049) which is light insensitive, and which responds irreversibly to temperature changes. Is the radical content of melanin related to its radical-scavenging or antioxidant activity? Although this issue is still widely unexplored, some studies have revealed that increasing the content of radicals in melanin either leads to an improved protection against X-ray irradiation of living keratinocytes [40] or to expansion of the antioxidant spectrum of melanin NP [76].

Results by Zareba et al. suggest, on the contrary, that reactive oxygen species generated by the photoexcited melanin contribute to overall phototoxicity [77]. We would like to stress that this study was carried out by exposing melanosomes to highly intense visible irradiation (4 mW mm^2^), while sun irradiance in the visible range can be estimated to be around 0.4 mW mm^2^, and thus about one order of magnitude less intense. Indeed, this choice was perfectly justified by the purpose of the authors, which was to induce melanosome photobleaching as a model of accelerated aging. A similar approach was taken by Zadlo et al., who demonstrated that the antioxidant activity of melanosomes against peroxidation of liposomal lipids induced by irradiation of Bengal Rose as a photosensitizer (this molecule is known to photosensitize the formation of singlet oxygen [78]) disappears when melanosomes are photobleached [79]. We would like to stress that photobleaching is a photoinduced process that leads to a strong modification of the chemical structure of melanin and melanosomes, and it occurs only under very harsh irradiation conditions. Consequently, it is almost negligible under unfocused solar light exposure. Chiarelli-Neto et al. reported that excitation of melanin in hair leads to the photosensitized formation of singlet oxygen, a strongly oxidizing ROS [80]. Also, in this case, high-intensity excitation was used; in particular, ^1^O_2_ phosphorescence was detected at 1270 nm upon excitation at 532 nm with a pulsed laser. Pulse duration was 5 ns and pulse energy 1 mJ corresponding to a power of 200 KW. Chiarelli-Neto et al. also demonstrated the possible damaging of skin cells, melanocytes, by singlet oxygen generated by melanin under either visible (532 nm) or UVA (355 nm) exposure [81]. Szewczyk et al. reported photosensitized generation of ROS (in particular, superoxide anion and singlet oxygen) upon irradiation of synthetic eumelanins, formed by autooxidation of DOPA, or enzymatic oxidation of 5,6-dihydroxyindole-2-carboxylic acid and synthetic pheomelanins obtained by enzymatic oxidation of 5-S-cysteinyldopa or 1:1 mixture of DOPA and cysteine [82]. We would like to stress that at 450 nm irradiation, the quantum yield of singlet oxygen generation is as low as 0.0001, and that melanins quenched singlet oxygen efficiently. Ito et al. reported that UVA radiation causes the oxidation of DHICA to indole-5,6-quinone-2carboxylic acid in eumelanin, which is then degraded to form a photodegraded, pyrrolic moiety and finally to yield free pyrrole-2,3,5-tricarboxylic acid. This photodegradation process leads to a decrease of the UV filtering ability of melanin [83]. Summarizing, some publications [77,78,79,80,81,82] suggest that irradiation of melanin may lead to the degradation of melanin itself through the photosensitized production of ROS. Although these results are very interesting, we would like to stress that investigation of the photodegradation of melanin (which occurs under intense, prolonged irradiation) is beyond the purposes of this review. On the other hand, these results, which in part disagree with the idea that light filtering and antioxidant activity can synergistically work in melanin, demonstrate how important it is to investigate the interaction between these two processes. In this framework, it has recently been reported that brief (five-minute) irradiation of 1,8-dihydroxynaphthalene allomelanin to sunlight leads to an increase in its radical-scavenging activity [84].

More generally, we can conclude that whether light irradiation may or may not lead to an enhancement of either the radical-scavenging or antioxidant activity of melanin is a complicated experimental issue that requires further extensive investigation.

## 6. Perspectives

The fundamental question we aim to answer in this paper is whether the antioxidant activity of melanin can be enhanced by light absorption and, hence, if light absorption and antioxidant activity may work synergistically in photoprotection. The best answer is that, at present, it is very difficult, and unusual, to perform antioxidant activity tests under irradiation.

**Figure 8:**
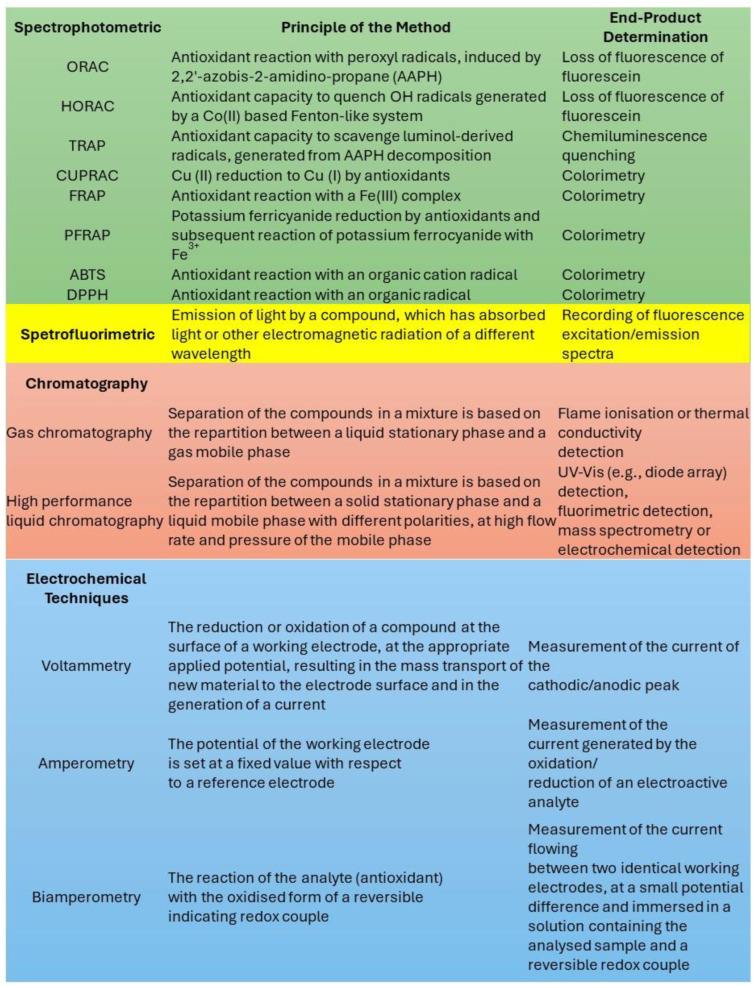
The most common methods used for the determination of antioxidant activity. Adapted from ref. [55].

As summarized in Figure 8, several analytical tests have been proposed for investigating antioxidant activity [55]; however, they are typically based on the spectrofluorimetric determination of some specific indicators (e.g., DPPH and fluorescein), which can be degraded upon light irradiation [55]. Moreover, these tests require an accurate control of the temperature, which can be altered by the irradiation of strongly colored species like melanin, due to the photothermal effect. We therefore suggest that tests for antioxidant activity should be redesigned to be performed under controlled irradiation, which requires the following:

*Correction for photodegradation of the probes.* Molecular probes like DPPH and fluorescein are known to undergo photodegradation upon irradiation. Hence, irradiation itself is expected to affect the results of conventional antioxidant activity tests. On the other hand, comparative tests may be exploited to counteract this effect. Reference samples should be irradiated to distinguish between the direct effect of the light on the probe and its effect on the antioxidant activity.

*Monitoring and control of the temperature.* Irradiation may produce temperature changes, e.g., because of the photothermal effect; hence, temperature requires accurate monitoring. The use of thermo-cameras may allow the identification of temperature inhomogeneity in the sample. Moreover, the temperature profiles should be used to perform reference experiments to correct for the temperature change.

## 7. Conclusions

Melanin is a natural photoprotective material. Artificial sunscreens mimic the photoprotective action of melanin, which is a combination of light filtering, mostly through light absorption, and antioxidant activity. Until now, the two mechanisms have been considered widely independent. Nevertheless, future research may prove that the antioxidant activity of melanin can be enhanced by light absorption. Confirming this hypothesis would demonstrate that the two photoprotective mechanisms, by nature, work synergistically. Presently, demonstrating this hypothesis is complicated by the absence of recognized tests for determining antioxidant activity under irradiation. We believe that making available and reliable this kind of measurement would provide a fundamental tool for the design and optimization of novel photoactive materials, suitable not only for photoprotection but also for phototherapy, energy conversion and storage, and environmental remediation.

## Abbreviations

The following abbreviations are used in this manuscript:

UV	Ultraviolet
NPs	Nanoparticles
ROS	Reactive oxygen species
NIR	Near infrared
HQ	Di-hydroxyquinones
SQ	Semiquinones
Q	Quinones
PDCA	Pyrrole-2,3-dicarboxylic acid
PDA	Polydopamine
UFTA	Ultrafast transient absorption
RONS	Reactive oxygen and nitrogen species
EPR	Electron paramagnetic resonance
CCR	Carbon centered radical
HAT	Hydrogen atom transfer
DPPH	2,2-diphenyl-1-picrylhydrazyl
